# Supplementary material for: Chronic Partial Sleep Deprivation Increased the Incidence of Atrial Fibrillation by Promoting Pulmonary Vein and Atrial Arrhythmogenesis in a Rodent Model
Source: Int J Mol Sci. 2024 Jul 11;25(14):7619. doi: 10.3390/ijms25147619 (PMC11277294; doi:10.3390/ijms25147619)
Supplement: Supplementary file 1 [file ijms-25-07619-s001.zip › ijms-3074455-supplementary.pdf]

Supplementary Material

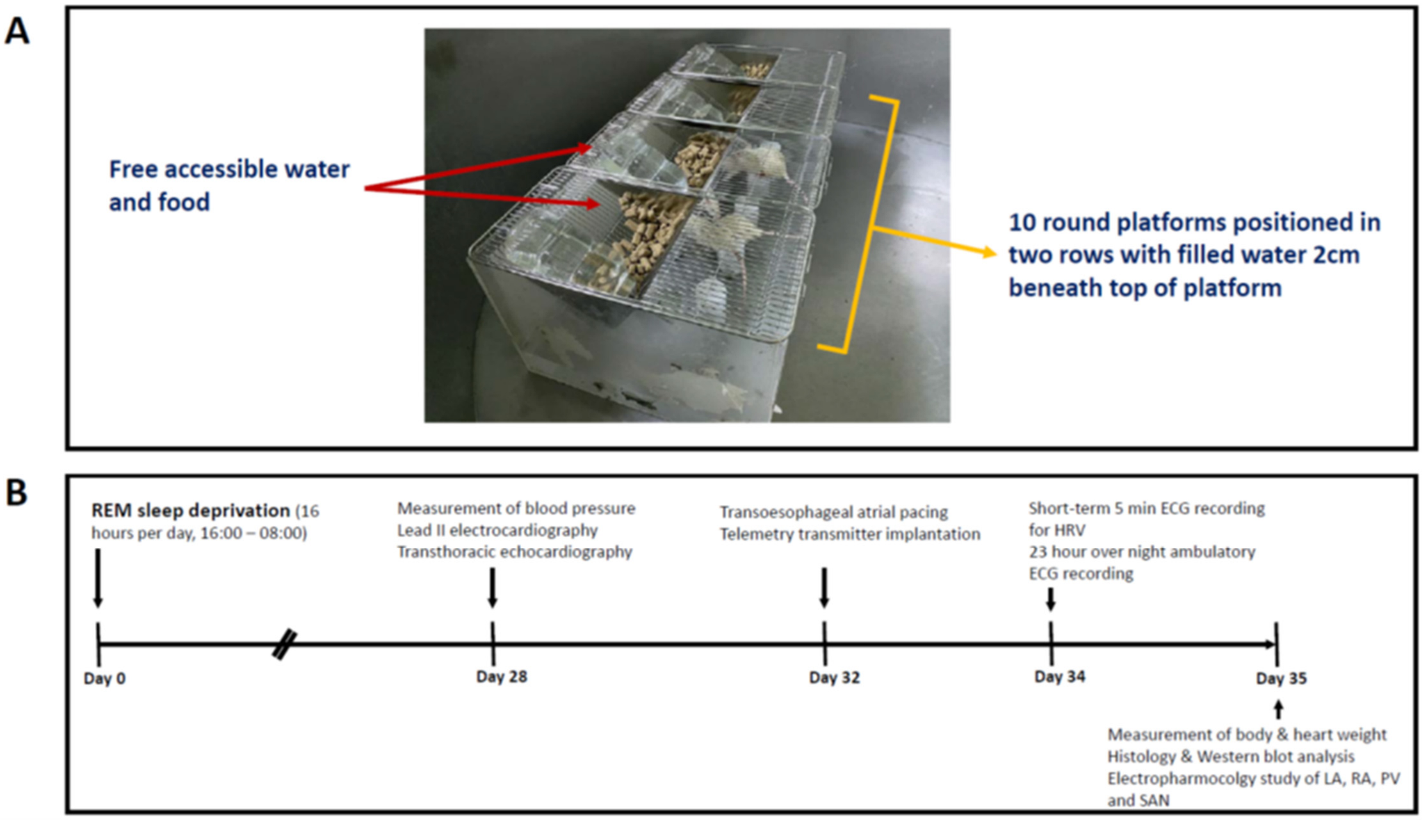

Supplementary Figure S1. Experimental design of the present study. (A) Photo illustration of water tank used to induce sleep deprivation in male Wistar rats. Freely movable rats upon circular platforms were demonstrated with water filled 2 cm below the platforms. (B) Graphic illustration demonstrating the experimental protocol. The rats in the sleep deprivation group were subjected to sleep deprivation for 16 hours per day (16:00-08:00) over 4 weeks. The experiment timeline was marked as corresponding days of sleep deprivation. Age-matched control rats will receive the same experiment.
